# Hormone Replacement Therapy, Likely Neither Angel Nor Demon

**DOI:** 10.1371/journal.pone.0138556

**Published:** 2015-09-18

**Authors:** Mitchell S. Wachtel, Shengping Yang, Sharmila Dissanaike, Julie A. Margenthaler

**Affiliations:** 1 Department of Pathology, Texas Tech University Health Sciences Center, Lubbock, Texas, United States of America; 2 Department of Surgery, Texas Tech University Health Sciences Center, Lubbock, Texas, United States of America; 3 Department of Surgery, Washington University School of Medicine, St. Louis, Missouri, United States of America; Tulane University Health Sciences Center, UNITED STATES

## Abstract

**Purpose:**

A decline in breast cancer incidence has been attributed to the reduction in hormone replacement therapy (HRT) prescriptions since the publication of the landmark WHIT paper in 2003. Concurrently, a relationship between HRT and cerebrovascular disease incidence has also been suggested. No generalized analysis of HRT prescription rates and breast cancer incidence rates that included more than seven years of data. We hypothesized that detailed analysis of SEER data would clarify the relationship between HRT use and breast cancer incidence. Given the large decline in HRT prescription rates uncovered, analyses of potential complications were also conducted, with the understanding that a small effect or one limited to a subpopulation, such as a single race, might not be detected.

**Methods:**

Incidence rates (per 100,000 women) and standard errors for ductal and lobular breast carcinomas, and endometrioid /endometrial carcinomas in women over 50 years were obtained from the Surveillance, Epidemiology, and End Results (SEER) database 1992–2012. From the Medical Expenditure Panel Survey 1996–2012 weighted counts and standard errors of hormone replacement therapy (HRT) prescriptions for women over 50 years were obtained. Using the National Hospital Discharge Survey (NHDS), 1996–2010 weighted counts and standard errors of femoral neck fractures, total hip replacements, acute myocardial infarctions, and cerebral infarctions were obtained for 50+ year men and women. Weighted counts and standard errors were divided by US census figures and multiplied by 100,000. Joinpoint regression was used to analyze rates.

**Main Results:**

Beginning 2001, HRT prescription rates dropped dramatically, 2001–2012 AAPC -14.9 (95% CI -17.4, -12.4). Breast cancer rates, which began to decline in 1999, increased after 2003; 2012 rates were similar to those seen in 2001 for both ductal, AAPC 0.1 (-0.4, 0.6) and lobular, AAPC 0.5 (-0.4, 1.5), carcinoma. Endometrial carcinoma rates increased, 2001–2012 AAPC 3.5 (3.1, 3.8), arguing against a negative effect of HRT discontinuation of endometrial carcinoma. Tests for parallelism failed to detect APC differences among genders for femoral neck fractures (*P* = 0.24), for total hip replacements (*P* = 0.11), for myocardial infarctions (*P* = 0.10), or for cerebral infarctions (*P* = 0.19), precluding any assignment of general effect on these disorders by HRT.

**Conclusions:**

Using SEER data, we demonstrated that changes in breast cancer rates cannot be explained by HRT prescription rate changes.

## Introduction

After a meta-analysis of epidemiological studies suggested that hormone replacement therapy (HRT) was a risk factor for breast cancer,[1] a prospective trial, the women’s health initiative randomized trial (WHIT), was conducted; results were abandoned after the data revealed a statistically significant difference in risk, reported in 2003.[2] A New England Journal of Medicine article in 2007 found a post 2003 decline in breast cancer incidence, which it deemed evidence of a confirmation of the findings of the 2003 WHIT paper.[3] Replies to this article suggested alternative conclusions.[4–6] A second report from WHIT suggested a connection between cerebrovascular accidents and HRT.[7] A third re-analysis of the data[8] was said to support the prior analysis, while suggesting that SEER data lent support to their results. Extensive analysis of SEER data, however, was lacking in that report. In addition, in the third report, for both the Estrogen+ Progestin (E+P) and the estrogen (E) alone trials, the treated groups had hazard ratios less than 1 (protective) for at least 2 years, which indicates that were HRT positively associated with breast cancer rate, the effect is unlikely to be immediate. Moreover, although potential contributions to changes as respects hip fractures and cardiovascular diseases were evaluated in a separate analyses of WHIT data,[9] no generalized analysis of these changes has been done as respects changes in HRT prescription rates.

This analysis aims at using population level data, to confirm/address: 1) is there reason to believe, as suggested by the third WHIT report,[8] that changes in breast cancer incidence rates reflect changes in HRT prescription rates? 2) is there reason to believe that changes in HRT rates had a positive or negative impact on changes in rates of hip fractures, hip replacements, myocardial infarctions or cerebrovascular accidents?

## Materials and Methods

The Institutional Review Board at Texas Tech University Health Sciences Center has in place a policy of not reviewing projects involving publicly available data; publicly available data for this study was obtained from the sources listed below, patient records/information for which had been anonymized and de-identified prior to analysis.

The Surveillance, Epidemiology, and End Results (SEER) Program of the National Cancer Institute (NCI) collects cancer incidence data from population-based cancer registries covering about a fourth of the US population.[10] SEER 13 included 1992–2012 data from the Atlanta, Connecticut, Detroit, Hawaii, Iowa, New Mexico, San Francisco-Oakland, Seattle-Puget Sound, Utah, Los Angeles, San Jose-Monterey, Rural Georgia, and the Alaska Native Tumor Registries.[11] The SEER*stat program[12] obtained from the SEER 13 registries database[13] 1992–2012 incidence rates and standard errors for women 50+ y, histologically proven invasive ductal carcinoma (ICD-O-3 8500/3) and lobular carcinoma (ICD-O-3 8520/3) of the breast and endometrioid carcinoma (ICD-0-3 8380/3) of the uterus. For ductal and separately for lobular carcinoma, incidence rates and standard errors were obtained for races White and Black, as well as age groups, 50–59 y, 60–69 y, 70–79 y, 80+ y; the selection of these decades was to parallel the WHIT study population. Rates per 100,000, their standard errors, and the associated raw data are supplied in Table A in S1 Table.

Following the review of a report from the Medical Expenditure Panel Survey,[14] a work order was issued to obtain estimates (weighted counts) and standard errors of hormone replacement therapy (HRT) prescriptions for 50+ y women from all available years, 1996–2012;[15] Therapeutic class and subclass were assigned to MEPS prescribed medicines using Multum Lexicon variables from Cerner Multum, Inc. MEPS prescribed medicines files were linked to the Multum Lexicon database to obtain therapeutic class and subclass variables. The first choice in the linking algorithm was chosen when assigning therapeutic classes and subclasses. For all years of data, the following was used to define hormone replacement therapy drugs: therapeutic class: Hormones; subclass: Sex Hormones; and sub subclasses: Estrogens, Progestins, and Sex Hormone Combinations. Rates for weighted counts and their standard errors were divided by US population values for women 50+ y as determined by the US Census and multiplied by 100,000 to obtain rates per 100,000 woman years and standard errors. Rates per 100,000, their standard errors, and the associated raw data are supplied in Table A in S1 Table.

The incidence of femoral neck fractures (ICD-9 820.8), total hip replacements (ICD-9 8151), acute myocardial infarctions (ICD-9 410.00–410.92), and cerebral infarctions (ICD-9 433.01, 433.11, 433.21, 433.31,433.81, 433.91, 434.01, 434.11, 434.91) was obtained via the National Hospital Discharge Survey[16] for all available years, 1996–2010, for both men and women. For each available year, weighted counts were obtained by a search for the ICD-9 codes for the condition/procedure of interest. Relative standard errors for each weighted count were calculated using the two parameters supplied for each year in the associated documentation, which were then multiplied by the weighted counts to yield standard errors. Weighted counts and their standard errors were divided by US population values for women and for men 50+ y as determined by the US Census and multiplied by 100,000 to obtain rates per 100,000 man|woman years. Rates per 100,000, their standard errors, and the associated raw data are supplied in Table A in S1 Table.

The Joinpoint Regression Program[17], with default settings, log linear regression, and grid search method calculated estimates, standard errors, and 95% confidence intervals for joinpoints, annual percentage changes (APC), and average annual percent changes (AAPC), as well as tests of parallelism was used. Tests of parallelism were performed on the sexes with reference to femoral neck fractures, total hip replacements, myocardial infarctions, and cerebrovascular accidents, as a lack of difference in APC among the genders would imply a lack of effect on those disorders from a large change in HRT prescription rates. Tests of parallelism were also performed between lobular and ductal carcinoma, to see if it would be reasonable to combine the two types of breast cancer, and for the age and racial subgroups of lobular and ductal carcinoma to see a) if there were differences between subgroup APC’s and overall APC’s, and b) to see if there were differences among the subgroups. Joinpoint estimated time periods, APC (95% CI), AAPC (95% CI), and P values are displayed in Table B in S1 Table. R 3.0.1[18] drew the figure. Null hypotheses were rejected when P < 0.05.

## Results

**Fig 1:**
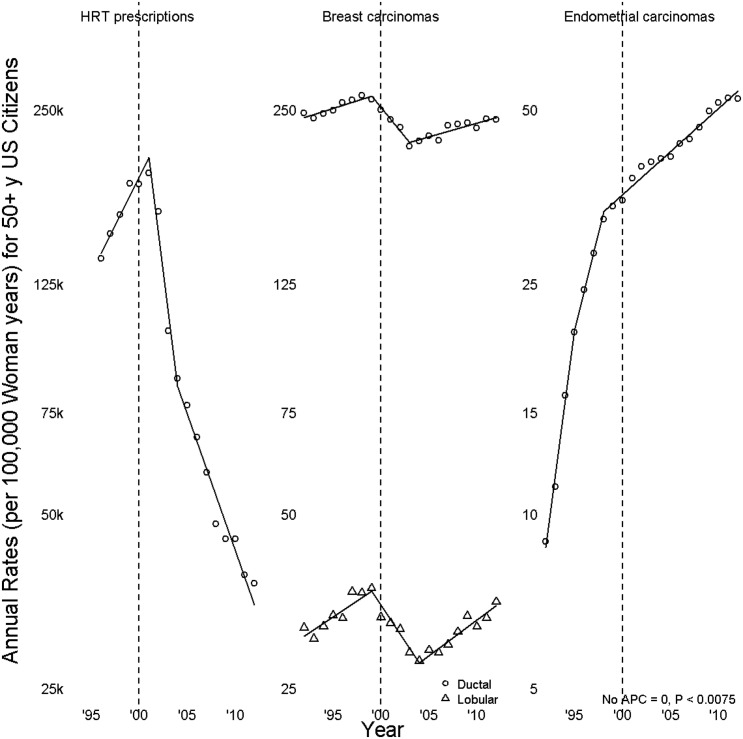
Hormone replacement therapy (HRT) prescriptions, breast carcinomas, and endometrial carcinomas.

**Fig 2:**
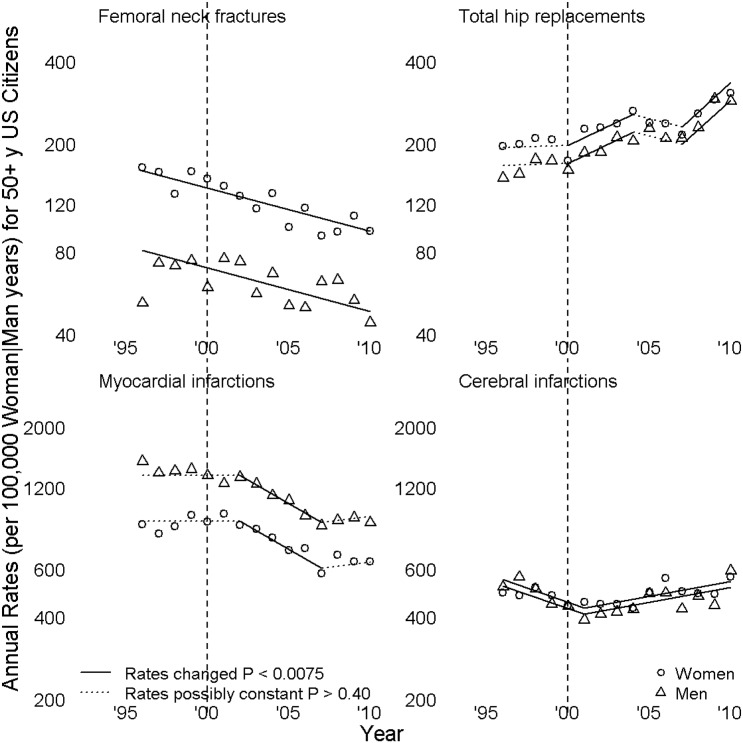
Femoral neck fractures, total hip replacements, myocardial infarctions, and cerebral infarctions.

**Fig 3:**
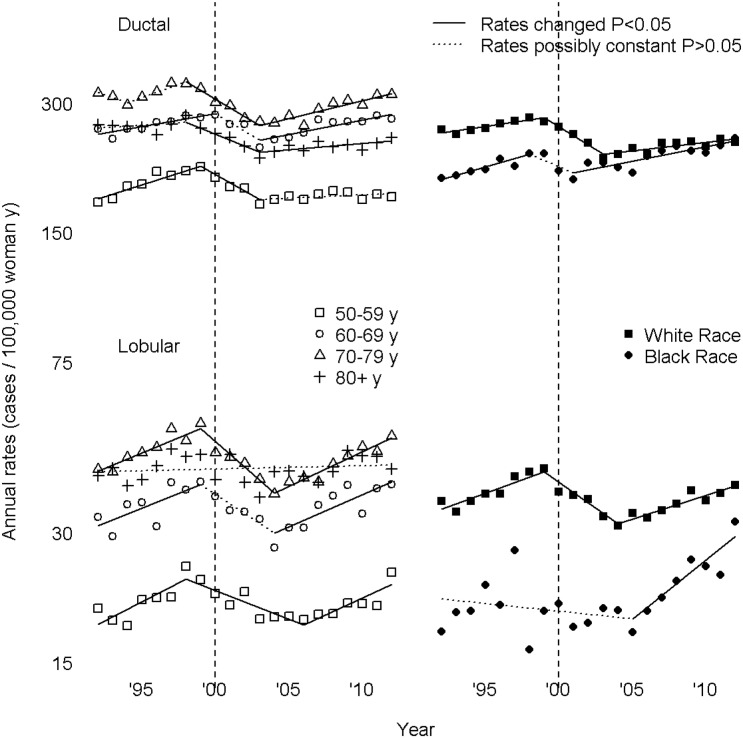
Age and Race Subgroups as Respects Ductal and Lobular Carcinomas of the Breast).

In Figs 1, 2 and 3, symbols lie at annual incidence rates. Line segment slopes are annual percent changes (APC), solid when not zero (rates changed), dotted when rates might be constant. Segments meet at joinpoints, points where APC was estimated to have changed; the left graph of Fig 1, of HRT prescriptions, had two joinpoints, a 2001 rate peak and a 2004 slowing of a decline in rates. Dashed vertical grey lines at 2000 enable the reader to count years before and after 2000 by noting the number of symbols between any point of interest and the grey line; periods of greatest interest near the turn of the century.

Fig 1 displays rates and APC for HRT prescriptions, for invasive breast cancer, and for endometrioid carcinoma. Breast cancer rates peaked in 1999, thereafter declining for ductal carcinoma, APC -4.5 (-6.8, -2.2), and lobular carcinoma, APC -5.6 (-8.3, -2.7). Rates increased for ductal carcinoma, APC 1.1 (0.7, 1.5), beginning 2003 and for lobular carcinoma, APC 2.9 (2.0, 3.9), beginning 2004. A test for parallelism showed that lobular carcinoma rates did not parallel ductal carcinoma rates (*P* = 0.002); that joinpoints and APC for lobular carcinoma were different from those for ductal carcinoma supports the differences seen in prior survival[19] and molecular[20, 21] analyses. Endometrioid carcinoma increased throughout the period of study; it did so at decreasing rates, with APC from 1992 until 1995 (1994, 1997) of 33.3 (23, 44.4) and, beginning 1998 (1997, 2012), of 3.5 (3.1, 3.8).

There was an extreme drop in HRT prescriptions beginning 2001 (95% CI 2000, 2002), which then slowed beginning 2004. The overall magnitude can be expressed either as an AAPC of -14.9 (95% CI -17.4, -12.4) or by saying that 2012 rates were 0.17 (0.12, 0.23) times those of 2001 rates. By contrast, for 2001–2012 rates for ductal carcinoma, AAPC 0.1 (-0.4, 0.6), and for lobular carcinoma, AAPC 0.5 (-0.4, 1.5), were not found to have changed. Because lobular and ductal carcinoma rates peaked in 1999, with the turning points of APR in 1999, breast cancer rate peaks were considered to have occurred prior to the HRT rate peak. Were HRT and breast cancer rates associated, then the incidence of breast cancer would most likely have been higher in 2000 and 2001 because of the 2001 HRT; given a lag effect (according to the 3^rd^ WHIT report, a less than 1 hazard ratio for the first two years since randomization, for both the E+P or E alone trials), breast cancer incidence should have been high even in 2002. However, Fig 1 does not show that type of relationship, raising questions as to its presence. After 2003, the slope of HRT prescription rate is a little flatter than that from 2001–2003, however, the absolute annual HRT prescription rate is still lower than that from 2001–2003. If HRT and breast cancer rates were fully correlated, then breast cancer rate should have been be lower for 2003–2010 than that from 2001–2003.

For endometrioid carcinoma rates, the 2001–2012 AAPC is the same as the 1999–2012 APC; 2012 rates were 1.46 (1.40, 1.51) times those of 2001 rates. The decline in HRT prescriptions is not reflected by changes seen in endometrioid carcinoma rates.

Fig 2 shows annual rates and APC for national hospital discharge survey data. Tests for parallelism failed to detect APC differences among genders for femoral neck fractures (*P* = 0.24), total hip replacements (*P* = 0.11), myocardial infarctions (*P* = 0.10), or cerebral infarctions (*P* = 0.19). The lack of a difference implies the effect of HRT prescriptions on these disorders cannot be very large at the level of a population of SEER size, since incidence rates of each condition for the sex that does not receive HRT (i.e. men) parallel those of the sex that did receive them. Femoral neck fracture rates decline throughout the study period, APC -3.6 (-4.7, -2.5); this is potentially explicable by the overall increment in total hip replacement rates, AAPC 4.1 (0.9, 7.2) given that total hip replacement precludes femoral neck fractures unless hip replacement is occasioned by the femoral neck fracture. Rates of myocardial infarction, AAPC -2.4 (-4.8, -0.03), decline over the entire period of study. Cerebral infarction rates first decline, 1996–2001 APC -4.6 (-7.8, -1.4), and then increase, 2001–2010 APC 2.5 (1.0, 4.0). Without taking into account the similarity of APC for stroke between men and women, it might be possible to argue HRT has a protective effect as respects cerebrovascular accidents; however because the 2001–2010 rate increase is similar for men and women, that conclusion would not be justified.

Tests for parallelism show different findings for ductal and lobular carcinoma as respects age groups. As compared with APC for the overall population, for ductal carcinoma no age subgroup has similar APC (*P* < 0.05, for each analysis); for lobular carcinoma, except for 60–60 y (*P* = 0.01), all age subgroups have similar APC (*P* > 0.05, for each analysis). As compared with APC for 50–59 y, for ductal carcinoma APC for 60–69 y (*P* < 0.001) and 70–79 y (*P* < 0.001) differ, while those for 80+ y are similar (*P* > 0.05). With respect to race, for both ductal and lobular carcinoma, APC for Whites and Blacks differ from that for the overall population and from one another (*P* < 0.05, for each analysis).

Fig 3 shows rates and APC by age and race subgroup for ductal and lobular carcinoma. If HRT prescription rate changes occur more for younger than for older women, one would expect to see any difference between 50–59 y and 60–69 y magnified for 70–79 y women. For 2003–2012 APC differences among decades show a trend indicating a larger increase among older women—50–59 y 0.4 (-0.2, 0.9), 60–69 y 1.5 (0.9, 2.2), 70–79 y 1.9 (1.2, 2.6). For preceding periods, no magnification is present—50–59 y, 1999–2003, -4.4 (-7.1, -1.5), 60–69 y, 2000–2003. -4.6 (-11.8, 3.1), 70–79 y, 1998–2003, -4.6 (-6.8, -2.4). For lobular carcinoma, results of parallelism tests do not support an age-related effect. Comparisons of the age groups originally studied by WHIT,[2, 8] shows no evidence of an age related effect. For both ductal and lobular carcinoma, the approach to equality between Black and White women in the last period of analysis cannot be explained on a general effect of HRT on breast cancer incidence. All subgroups showed rate peaks before 2001, except Black women with ductal carcinoma, which never showed a rate peak.

## Conclusions and Discussion

The epidemiological studies evaluated in 1997[1] found that women who had used HRT beginning at age 50 had an extra 2 (95% CI 1, 3) breast cancers per 1,000 women after five years of use, an extra 6 (3, 9) cancers after ten years use, and an extra 12 (5, 20) cancers after 15 years of use. A prospective randomized trial, the Women’s health Initiative Randomized Trial (WHIT) reported in 2003 a greater risk of breast cancer among patients being given estrogen progesterone HRT than those who had not;[2] the study was abandoned after an average of 5.6 y and showed an increase in risk of breast cancer, HR 1.24 (1.01, 1.54).

A 2007 report indicated that there had been a decline in breast cancer incidence in 2003, and related this decline to discontinuation of post-menopausal hormone replacement therapy (HRT).[3] Several limitations of generalized conclusions from this paper’s data were provisioned in letters to the editor: Canadian breast cancer incidences peaked before 2003;[4] Scandinavian rates of HRT use did not correlate with breast cancer incidence;[5] and estrogen positive breast cancer rates were noted in the US to have peaked in 1999.[6]

A later trial of estrogen only HRT versus placebo by WHIT,[7] showed a long-term risk reduction of breast cancer accorded to estrogen only HRT, HR 0.77 (0.62, 0.95) that was opposite and nearly equivalent in magnitude to the differences documented in the prior studies. The trial was abandoned, however, because of an increased number of cerebrovascular accidents in the treatment arm;[9] no deleterious effect as respects myocardial infarction or hip fractures was seen.

Provided the limitations of generalized analyses and survey data are taken into account, these findings can be evaluated by examining changes in overall rates of the varying disorders. The MEPS survey only included household survey data, was not verified with claims data, only included outpatient prescription medicines in an outpatient setting, included both refills and original prescriptions, and did not distinguish types of HRT. The NIHS survey data was calculated from codes submitted from billing, not from diagnoses submitted from a uniform population of health care providers, did not include nursing home stays or office visits, and did not distinguish first or second episodes of events. SEER data were from diagnoses rendered by community and academic pathologists without central review, did not distinguish first from second malignancies, and did not supply information as to the health status in general of the associated population.

Because the databases are unlinked, no stratification as respects covariates could be attempted. This disadvantage, however, is balanced by two factors. Because men did not receive HRT, when the genders show parallel rates, then HRT cannot be plausibly said to explain changes in those rates. Because breast cancer is so rare in men,[22] assessing parallelism between gender rates was not a viable option. The results of parallelism tests indicate that, given the extraordinary drop in HRT prescriptions, there is little or no reason to believe that such drops influenced rates of femoral neck fractures, total hip replacements, myocardial infarctions, or cerebral infarctions. Data was not available as respects the different formulations of HRT; because the drop in HRT rates was so large and because estrogen-only formulations were said to provide long-term protection against breast cancer,[7] the separate analysis would not have contributed to an understanding of why breast cancer rates increased after 2003.

The second advantage of the current analysis lies in the assessment of potential bad outcomes of a drug or drug combination that shows marked increases or decreases in prescription rates. As shown, none of the evaluated factors could be said to have been influenced by HRT. Endometrioid carcinoma rates increased over the entire period of study, implying that the HRT formulations used during the period of study did not play a prominent role. Because the genders did not differ as respects the remaining factors, HRT cannot be said to have exhibited a large influence on them; neither can it be said, from this analysis of disease markers, that changes in other potential risk factors were different for men than for women.

Because the results conflict with the conclusions of both major WHIT evaluations,[2, 8] some discussion is warranted specifically with respect to those articles. First, Fig 1 of the first report[2] shows crossed cumulative incidence curves at 3.5 y; the crossings invalidate Cox regressions performed in both reports.[2, 8] Second, rates were not stable after 2003, as indicated by the second WHIT report;[8] they increased until the end of the study period. Third, although the second report states that in “SEER, a substantial drop in breast cancer in 2003 was coincident with the decrease in menopausal hormone therapy in the United States,”[8] it fails to address the issue previously noted[6]—breast cancer rates peaked before the 2003 study. The current analysis further substantiates that claim: not only did this analysis show that rates for invasive ductal carcinoma and invasive lobular carcinoma peaked before 2003, it showed that all racial and age subgroups that had peaks did so before the 2001 peak in HRT prescription rates.

Approximately 85% of women who participated in the WHIT trial are white, which is substantially higher than the 64% white women in the SEER population. Based on the parallelism test, for both ductal and lobular carcinoma, Whites and Blacks differ significantly from each other; thus some of the discrepancies between the WHIT trial and SEER based studies can be partially explained by the differences in patient characteristics.

The cause of the decline remains unsettled. One possible contribution to a decline might be chemoprevention, first recommended as respects Tamoxifen, for general use in 1998. The only database available is a survey of patients, not physicians as to Tamoxifen therapy. A 2010 report showed that between 2000, 0.2% (0.13, 0.31%) and 2005, 0.8% (0.03–0.17%), only a small fraction of women had taken the drug without definite differences over time.[23] Because the FDA did not grant approval for this use of Tamoxifen until 2007, the quality of the data before and after that date are dissimilar, precluding a valid analysis of tamoxifen use over the period in question. The 2012 analysis showed that in 2010, 0.3% (0.001, 0.15%) of women 35–79 y were taking Tamoxifen chemoprevention, which does not differ from the prior two results.[24] Thus, although chemoprevention might have contributed to the observed decline in breast cancer rates, it is very unlikely that it would have accounted for it entirely. Should the factor be analyzed in the future, complexities would include the additional chemoprevention strategies that have since been adopted.[25–29] Such an analysis, of course, unless it showed a decline in chemoprevention, would be unable to explain the increase in breast cancer rates seen in this study.

HRT prescription rates showed a dramatic drop from its 2001 peak. This marked decline was not reflected in any of the other analyzed rates. Rates for both lobular and ductal breast cancer showed a decline that began before the HRT peak, but then showed an increase, such that by 2012 rates were similar to those seen in 2001, indicating there is no clear relationship between HRT and breast cancer. Endometrial carcinoma rates increased, the opposite of what would have been expected had exogenous hormones been of importance in that disease. Rates for women for femoral neck fractures, total hip replacements, myocardial infarctions, and cerebral infarctions, paralleled those of men, who received no HRT prescriptions, As mentioned before, this lack of difference among the sexes argues against an effect on these disorders as respects HRT; however, owing to the nature of population level data, a small effect or one limited to a subgroup of the population might not have been detected.

## Supporting Information

S1 Table Raw data and joinpoint calculated estimates and standard errors. (DOCX)
