# Supplementary material for: Hormone Replacement Therapy, Likely Neither Angel Nor Demon
Source: PLoS One. 2015 Sep 18;10(9):e0138556. doi: 10.1371/journal.pone.0138556 (PMC4575090; doi:10.1371/journal.pone.0138556)
Supplement: S1 Table — (DOCX) [file pone.0138556.s001.docx]

Table A. Raw data.

| **Data from surveys** | |  |  |
| --- | --- | --- | --- |
| Year | Rate (SE) (/100,000) | Weighted Count (SE) | Population |
| **Medical Expenditure Panel Survey** | | |  |
| Hormone replacement therapy prescriptions | | |  |
| 1996 | 138074.8 (7271.1) | 53536283 (2819255) | 38773395 |
| 1997 | 152360.9 (7188.3) | 60631731 (2860571) | 39794805 |
| 1998 | 164537.7 (9031.6) | 66896401 (3671996) | 40657194 |
| 1999 | 186210.2 (9720.6) | 77315719 (4036056) | 41520664 |
| 2000 | 185826.3 (13248.1) | 78782969 (5616676) | 42396028 |
| 2001 | 193940.8 (8824.4) | 83916217 (3818238) | 43268987 |
| 2002 | 166349.3 (6872.2) | 73499259 (3036407) | 44183678 |
| 2003 | 103614.9 (5040) | 46801221 (2276494) | 45168407 |
| 2004 | 85695.2 (5100.5) | 39592080 (2356483) | 46201059 |
| 2005 | 76926.1 (4851.8) | 36407351 (2296261) | 47327710 |
| 2006 | 67878.6 (4306) | 32900097 (2087080) | 48469036 |
| 2007 | 58928.8 (4073) | 29276443 (2023528) | 49681022 |
| 2008 | 48021 (3567.5) | 24450366 (1816425) | 50916029 |
| 2009 | 45252.9 (3598.6) | 23598757 (1876613) | 52148572 |
| 2010 | 45253 (3913.7) | 24169891 (2090352) | 53410602 |
| 2011 | 39178.9 (3574.7) | 21410634 (1953499) | 54648357 |
| 2012 | 37915.2 (3563.1) | 21161465 (1988675) | 55812633 |
| **National Hospital Discharge Survey** | | |  |
| Femoral Neck Fractures | |  |  |
| Women |  |  |  |
| 1996 | 163.7 (25) | 63461 (9705.9) | 38773395 |
| 1997 | 157.4 (22) | 62641 (8735.3) | 39794805 |
| 1998 | 130.9 (11.7) | 53224 (4766.4) | 40657194 |
| 1999 | 158.1 (14.8) | 65653 (6138.4) | 41520664 |
| 2000 | 148.8 (12.1) | 63099 (5117) | 42396028 |
| 2001 | 140.3 (11.8) | 60701 (5115.9) | 43268987 |
| 2002 | 128.5 (12.1) | 56777 (5330.8) | 44183678 |
| 2003 | 115.7 (11.9) | 52266 (5385.3) | 45168407 |
| 2004 | 131.4 (13.3) | 60690 (6155.6) | 46201059 |
| 2005 | 98.7 (9.9) | 46698 (4661.6) | 47327710 |
| 2006 | 116.1 (12) | 56291 (5835) | 48469036 |
| 2007 | 91.8 (9.5) | 45587 (4699.1) | 49681022 |
| 2008 | 95.1 (16.6) | 48437 (8461.2) | 50916029 |
| 2009 | 109.1 (17.4) | 56876 (9060) | 52148572 |
| 2010 | 95.8 (15.7) | 51168 (8364.6) | 53410602 |
| Men |  |  |  |
| 1996 | 52 (14.2) | 16199 (4411.1) | 31149079 |
| 1997 | 72.9 (17.7) | 23461 (5689.3) | 32196935 |
| 1998 | 71.2 (8.8) | 23568 (2912.2) | 33090614 |
| 1999 | 74.4 (10.1) | 25284 (3436.2) | 33978815 |
| 2000 | 59.4 (9.6) | 20705 (3352.2) | 34882053 |
| 2001 | 75.8 (9.4) | 27159 (3350.8) | 35813884 |
| 2002 | 73.8 (9.3) | 27159 (3402.3) | 36781767 |
| 2003 | 56.2 (8.1) | 21248 (3079) | 37801345 |
| 2004 | 66.8 (8.1) | 25963 (3139.6) | 38872232 |
| 2005 | 50.9 (6.8) | 20364 (2713.9) | 40042318 |
| 2006 | 50 (7.2) | 20608 (2980.1) | 41196057 |
| 2007 | 62.3 (7.8) | 26422 (3307.4) | 42413301 |
| 2008 | 63.1 (12.4) | 27543 (5434.6) | 43658129 |
| 2009 | 53.2 (11.2) | 23858 (5035.3) | 44885046 |
| 2010 | 44 (9.9) | 20328 (4565.7) | 46158693 |
| Total Hip Replacements | |  |  |
| Women |  |  |  |
| 1996 | 196.5 (16.6) | 76199 (6432.7) | 38773395 |
| 1997 | 199.3 (15.1) | 79321 (6014.1) | 39794805 |
| 1998 | 210.3 (16.8) | 85502 (6844) | 40657194 |
| 1999 | 207 (17.6) | 85961 (7291.4) | 41520664 |
| 2000 | 173.7 (14.4) | 73662 (6112.5) | 42396028 |
| 2001 | 227.1 (18.6) | 98262 (8028) | 43268987 |
| 2002 | 229.8 (17.3) | 101555 (7660.8) | 44183678 |
| 2003 | 237 (20.4) | 107038 (9198.2) | 45168407 |
| 2004 | 264.3 (25.4) | 122097 (11739.9) | 46201059 |
| 2005 | 239.2 (18.4) | 113216 (8699.6) | 47327710 |
| 2006 | 237.5 (19.5) | 115097 (9449) | 48469036 |
| 2007 | 216 (18) | 107296 (8929.6) | 49681022 |
| 2008 | 258 (36.1) | 131341 (18390.4) | 50916029 |
| 2009 | 290.8 (40.2) | 151637 (20968.8) | 52148572 |
| 2010 | 307.8 (48.2) | 164410 (25759.1) | 53410602 |
| Men |  |  |  |
| 1996 | 150 (15.4) | 46723 (4798.9) | 31149079 |
| 1997 | 155 (15.7) | 49914 (5065.1) | 32196935 |
| 1998 | 175.3 (16.6) | 57994 (5479.8) | 33090614 |
| 1999 | 173.2 (16.7) | 58842 (5660.7) | 33978815 |
| 2000 | 160.1 (15.8) | 55839 (5521.1) | 34882053 |
| 2001 | 185.2 (16.2) | 66345 (5802.4) | 35813884 |
| 2002 | 186.7 (16.2) | 68656 (5970) | 36781767 |
| 2003 | 211.3 (19.1) | 79884 (7217.3) | 37801345 |
| 2004 | 204.6 (18.5) | 79528 (7194.8) | 38872232 |
| 2005 | 227.7 (18.6) | 91168 (7430.1) | 40042318 |
| 2006 | 209.3 (19.1) | 86232 (7878.7) | 41196057 |
| 2007 | 209.5 (18.7) | 88846 (7935.9) | 42413301 |
| 2008 | 229.5 (33.2) | 100207 (14505) | 43658129 |
| 2009 | 292.4 (40.6) | 131243 (18232.8) | 44885046 |
| 2010 | 287.7 (41.3) | 132814 (19057.4) | 46158693 |
| Acute Myocardial Infarctions | |  |  |
| Women |  |  |  |
| 1996 | 874.9 (69) | 339237 (26739.7) | 38773395 |
| 1997 | 809.3 (61.7) | 322042 (24552.1) | 39794805 |
| 1998 | 862.9 (41.8) | 350841 (16986.1) | 40657194 |
| 1999 | 951 (64.5) | 394841 (26767.4) | 41520664 |
| 2000 | 897.4 (48.4) | 380468 (20509.3) | 42396028 |
| 2001 | 959.6 (51.3) | 415198 (22203) | 43268987 |
| 2002 | 871.2 (53.9) | 384945 (23803.3) | 44183678 |
| 2003 | 843.1 (50.9) | 380832 (22975.9) | 45168407 |
| 2004 | 784.5 (55.9) | 362444 (25827.3) | 46201059 |
| 2005 | 705.3 (42.3) | 333823 (20017.8) | 47327710 |
| 2006 | 716.6 (54.6) | 347305 (26481.5) | 48469036 |
| 2007 | 580.1 (34.9) | 288185 (17333.8) | 49681022 |
| 2008 | 678.8 (88.7) | 345634 (45147.8) | 50916029 |
| 2009 | 640.2 (80.2) | 333852 (41816.8) | 52148572 |
| 2010 | 640 (80.7) | 341806 (43106.4) | 53410602 |
| Men |  |  |  |
| 1996 | 1493.3 (136.2) | 465138 (42415.6) | 31149079 |
| 1997 | 1355.2 (104.9) | 436346 (33774.1) | 32196935 |
| 1998 | 1379.3 (97.9) | 456427 (32405.9) | 33090614 |
| 1999 | 1396.9 (97.4) | 474647 (33085.5) | 33978815 |
| 2000 | 1324.3 (96.2) | 461940 (33554.9) | 34882053 |
| 2001 | 1238.6 (69.1) | 443586 (24748.5) | 35813884 |
| 2002 | 1305.9 (66.3) | 480336 (24379.7) | 36781767 |
| 2003 | 1236.2 (70.6) | 467318 (26701.4) | 37801345 |
| 2004 | 1118.6 (67.5) | 434833 (26225.8) | 38872232 |
| 2005 | 1073.8 (60.1) | 429968 (24082.2) | 40042318 |
| 2006 | 945 (53.6) | 389296 (22087.1) | 41196057 |
| 2007 | 868.4 (52.4) | 368310 (22211.4) | 42413301 |
| 2008 | 907 (122.6) | 395993 (53544.9) | 43658129 |
| 2009 | 927.9 (118.9) | 416494 (53352.7) | 44885046 |
| 2010 | 889.9 (113.9) | 410779 (52563.5) | 46158693 |
| Cerebral Infarctions | |  |  |
| Women |  |  |  |
| 1996 | 409.5 (28.6) | 158768 (11075.3) | 38773395 |
| 1997 | 415.2 (25.5) | 165216 (10163.3) | 39794805 |
| 1998 | 404.6 (27.3) | 164496 (11103.5) | 40657194 |
| 1999 | 389.9 (29) | 161889 (12058.5) | 41520664 |
| 2000 | 341 (23.9) | 144562 (10128.5) | 42396028 |
| 2001 | 348.3 (26.1) | 150702 (11300.3) | 43268987 |
| 2002 | 344.6 (23.4) | 152236 (10330.8) | 44183678 |
| 2003 | 328.5 (26.7) | 148393 (12055.7) | 45168407 |
| 2004 | 338 (31.5) | 156148 (14543.5) | 46201059 |
| 2005 | 272.8 (20.4) | 129126 (9644.5) | 47327710 |
| 2006 | 284.2 (22.5) | 137761 (10899.2) | 48469036 |
| 2007 | 269.6 (21.6) | 133925 (10710.9) | 49681022 |
| 2008 | 273.7 (38.1) | 139377 (19388.5) | 50916029 |
| 2009 | 239.6 (33.9) | 124923 (17668.4) | 52148572 |
| 2010 | 278.5 (44.2) | 148774 (23584.5) | 53410602 |
| Men |  |  |  |
| 1996 | 332.4 (27.1) | 103551 (8453.3) | 31149079 |
| 1997 | 308 (24.9) | 99160 (8003.3) | 32196935 |
| 1998 | 279 (23.2) | 92334 (7670.1) | 33090614 |
| 1999 | 276.2 (23.3) | 93833 (7904) | 33978815 |
| 2000 | 285.1 (24.8) | 99434 (8634.6) | 34882053 |
| 2001 | 289.6 (22.3) | 103700 (8001.7) | 35813884 |
| 2002 | 283.3 (21.1) | 104200 (7759.1) | 36781767 |
| 2003 | 285.1 (23.9) | 107759 (9016.1) | 37801345 |
| 2004 | 242.5 (21) | 94282 (8160) | 38872232 |
| 2005 | 254.5 (20.2) | 101892 (8069.8) | 40042318 |
| 2006 | 220.3 (19.8) | 90774 (8173.3) | 41196057 |
| 2007 | 225.6 (19.9) | 95665 (8442) | 42413301 |
| 2008 | 259.9 (37) | 113466 (16165.6) | 43658129 |
| 2009 | 247.3 (35.1) | 111006 (15745.7) | 44885046 |
| 2010 | 224.6 (33.5) | 103689 (15444.9) | 46158693 |
| **Data from SEER 13-Regs** | |  |  |
| Year | Rate (SE) (/100,000) | Count | Population |
| Ductal Carcinoma of Breast | |  |  |
| 1992 | 246.5 (2.3) | 11353 | 4605645 |
| 1993 | 241.3 (2.3) | 11305 | 4684207 |
| 1994 | 245.9 (2.3) | 11662 | 4742583 |
| 1995 | 249.2 (2.3) | 11984 | 4809406 |
| 1996 | 256.8 (2.3) | 12511 | 4872302 |
| 1997 | 259.5 (2.3) | 12992 | 5007151 |
| 1998 | 264.6 (2.3) | 13551 | 5121456 |
| 1999 | 260.3 (2.2) | 13651 | 5244436 |
| 2000 | 249.8 (2.2) | 13403 | 5365165 |
| 2001 | 240.3 (2.1) | 13175 | 5483539 |
| 2002 | 233.1 (2) | 13062 | 5602644 |
| 2003 | 215.8 (1.9) | 12365 | 5730764 |
| 2004 | 220.3 (1.9) | 12912 | 5860116 |
| 2005 | 225.1 (1.9) | 13512 | 6001854 |
| 2006 | 221.3 (1.9) | 13597 | 6143196 |
| 2007 | 234.7 (1.9) | 14784 | 6298314 |
| 2008 | 235.8 (1.9) | 15227 | 6458452 |
| 2009 | 236.9 (1.9) | 15698 | 6626585 |
| 2010 | 232 (1.8) | 15775 | 6799933 |
| 2011 | 240.9 (1.9) | 16774 | 6962872 |
| 2012 | 239.9 (1.8) | 17084 | 7120060 |
| 50-59 y |  |  |  |
| 1992 | 176.9 (3.4) | 2742 | 1550315 |
| 1993 | 179.9 (3.3) | 2896 | 1609484 |
| 1994 | 192 (3.4) | 3184 | 1658325 |
| 1995 | 194.5 (3.4) | 3311 | 1702358 |
| 1996 | 208.3 (3.5) | 3639 | 1747392 |
| 1997 | 203.4 (3.3) | 3798 | 1867254 |
| 1998 | 208.9 (3.3) | 4099 | 1962150 |
| 1999 | 213 (3.2) | 4375 | 2054009 |
| 2000 | 201.2 (3.1) | 4319 | 2146103 |
| 2001 | 192 (2.9) | 4293 | 2236504 |
| 2002 | 190.2 (2.9) | 4410 | 2318999 |
| 2003 | 174.8 (2.7) | 4173 | 2387640 |
| 2004 | 179 (2.7) | 4417 | 2467192 |
| 2005 | 182.6 (2.7) | 4664 | 2553940 |
| 2006 | 178.9 (2.6) | 4724 | 2641145 |
| 2007 | 184.1 (2.6) | 4938 | 2682250 |
| 2008 | 187.3 (2.6) | 5126 | 2737442 |
| 2009 | 185.9 (2.6) | 5191 | 2791781 |
| 2010 | 179.3 (2.5) | 5104 | 2845990 |
| 2011 | 184.4 (2.5) | 5328 | 2888813 |
| 2012 | 181.9 (2.5) | 5323 | 2926706 |
| 60-69 y |  |  |  |
| 1992 | 261.7 (4.3) | 3622 | 1383959 |
| 1993 | 247.4 (4.3) | 3381 | 1366505 |
| 1994 | 261.8 (4.4) | 3521 | 1344859 |
| 1995 | 261.4 (4.4) | 3485 | 1333223 |
| 1996 | 270.5 (4.5) | 3581 | 1323631 |
| 1997 | 271.4 (4.5) | 3572 | 1316029 |
| 1998 | 281.1 (4.6) | 3699 | 1315830 |
| 1999 | 277.5 (4.6) | 3668 | 1321886 |
| 2000 | 282 (4.6) | 3748 | 1329284 |
| 2001 | 268.2 (4.5) | 3607 | 1344762 |
| 2002 | 267.8 (4.4) | 3679 | 1373648 |
| 2003 | 236.4 (4.1) | 3369 | 1424945 |
| 2004 | 246.7 (4.1) | 3632 | 1472476 |
| 2005 | 249.3 (4.1) | 3778 | 1515469 |
| 2006 | 256.1 (4.1) | 4000 | 1561719 |
| 2007 | 275 (4.1) | 4576 | 1664157 |
| 2008 | 271.1 (3.9) | 4753 | 1753471 |
| 2009 | 272.1 (3.8) | 5024 | 1846077 |
| 2010 | 271.5 (3.7) | 5267 | 1940255 |
| 2011 | 280.7 (3.7) | 5693 | 2028374 |
| 2012 | 275.4 (3.6) | 5809 | 2109458 |
| 70-79 y |  |  |  |
| 1992 | 316.6 (5.5) | 3338 | 1054285 |
| 1993 | 310.9 (5.4) | 3329 | 1070659 |
| 1994 | 296.7 (5.2) | 3219 | 1084764 |
| 1995 | 309.8 (5.3) | 3408 | 1100088 |
| 1996 | 318.9 (5.4) | 3541 | 1110431 |
| 1997 | 333.6 (5.5) | 3731 | 1118478 |
| 1998 | 332.1 (5.4) | 3734 | 1124313 |
| 1999 | 324.5 (5.4) | 3673 | 1131974 |
| 2000 | 300.7 (5.1) | 3418 | 1136769 |
| 2001 | 296 (5.1) | 3347 | 1130692 |
| 2002 | 275.9 (5) | 3092 | 1120518 |
| 2003 | 271.7 (4.9) | 3019 | 1111225 |
| 2004 | 269.1 (5) | 2954 | 1097556 |
| 2005 | 279.9 (5.1) | 3052 | 1090452 |
| 2006 | 265.8 (5) | 2881 | 1083724 |
| 2007 | 290.4 (5.2) | 3140 | 1081369 |
| 2008 | 300 (5.3) | 3255 | 1084843 |
| 2009 | 304.6 (5.3) | 3335 | 1094818 |
| 2010 | 297 (5.2) | 3286 | 1106578 |
| 2011 | 312.8 (5.3) | 3524 | 1126659 |
| 2012 | 314.2 (5.2) | 3634 | 1156443 |
| 80+ y |  |  |  |
| 1992 | 267.4 (6.6) | 1650 | 617086 |
| 1993 | 266 (6.5) | 1696 | 637559 |
| 1994 | 264.7 (6.4) | 1733 | 654635 |
| 1995 | 264.1 (6.3) | 1779 | 673737 |
| 1996 | 253.3 (6.1) | 1750 | 690848 |
| 1997 | 267.7 (6.2) | 1888 | 705390 |
| 1998 | 280.7 (6.2) | 2019 | 719163 |
| 1999 | 262.4 (6) | 1933 | 736567 |
| 2000 | 254.6 (5.8) | 1917 | 753009 |
| 2001 | 249.9 (5.7) | 1928 | 771581 |
| 2002 | 238 (5.5) | 1879 | 789479 |
| 2003 | 223.4 (5.3) | 1803 | 806954 |
| 2004 | 231.5 (5.3) | 1905 | 822892 |
| 2005 | 239.4 (5.3) | 2016 | 841993 |
| 2006 | 232.4 (5.2) | 1991 | 856608 |
| 2007 | 244.2 (5.3) | 2126 | 870538 |
| 2008 | 237.1 (5.2) | 2093 | 882696 |
| 2009 | 240 (5.2) | 2145 | 893909 |
| 2010 | 233.5 (5.1) | 2118 | 907110 |
| 2011 | 242.4 (5.1) | 2228 | 919026 |
| 2012 | 249.5 (5.2) | 2314 | 927453 |
| White women | |  |  |
| 1992 | 260.1 (2.6) | 9876 | 3797105 |
| 1993 | 254.1 (2.6) | 9775 | 3847020 |
| 1994 | 259.6 (2.6) | 10066 | 3876931 |
| 1995 | 262.6 (2.6) | 10276 | 3913085 |
| 1996 | 269.4 (2.6) | 10628 | 3945020 |
| 1997 | 272.5 (2.6) | 11002 | 4038125 |
| 1998 | 277.7 (2.6) | 11414 | 4109505 |
| 1999 | 272.2 (2.6) | 11397 | 4186570 |
| 2000 | 263.9 (2.5) | 11239 | 4258336 |
| 2001 | 253.7 (2.4) | 10984 | 4328972 |
| 2002 | 242.5 (2.3) | 10671 | 4400051 |
| 2003 | 223.1 (2.2) | 9992 | 4477858 |
| 2004 | 228.6 (2.2) | 10415 | 4556511 |
| 2005 | 235.5 (2.3) | 10933 | 4642997 |
| 2006 | 228 (2.2) | 10779 | 4728648 |
| 2007 | 242.7 (2.2) | 11715 | 4826625 |
| 2008 | 242.7 (2.2) | 11964 | 4929952 |
| 2009 | 244.4 (2.2) | 12304 | 5035115 |
| 2010 | 238.4 (2.2) | 12265 | 5145653 |
| 2011 | 247.6 (2.2) | 12999 | 5251056 |
| 2012 | 243.8 (2.1) | 13039 | 5349113 |
| Black women | |  |  |
| 1992 | 200.9 (7.1) | 794 | 395275 |
| 1993 | 203.8 (7.1) | 820 | 402427 |
| 1994 | 207.7 (7.1) | 851 | 409741 |
| 1995 | 210.3 (7.1) | 879 | 417971 |
| 1996 | 222.4 (7.2) | 947 | 425733 |
| 1997 | 214.2 (7) | 941 | 439289 |
| 1998 | 229.5 (7.1) | 1041 | 453667 |
| 1999 | 229.4 (7) | 1078 | 469920 |
| 2000 | 208.9 (6.6) | 1015 | 485765 |
| 2001 | 199.3 (6.3) | 1000 | 501720 |
| 2002 | 218.2 (6.5) | 1129 | 517412 |
| 2003 | 217.6 (6.4) | 1161 | 533564 |
| 2004 | 212.8 (6.2) | 1169 | 549419 |
| 2005 | 206.5 (6) | 1172 | 567539 |
| 2006 | 225.6 (6.2) | 1325 | 587434 |
| 2007 | 232.7 (6.2) | 1413 | 607234 |
| 2008 | 238.4 (6.2) | 1491 | 625438 |
| 2009 | 232.2 (6) | 1509 | 649775 |
| 2010 | 229.9 (5.8) | 1546 | 672454 |
| 2011 | 238.9 (5.9) | 1656 | 693048 |
| 2012 | 248.8 (5.9) | 1778 | 714539 |
| Lobular Carcioma of Breast | |  |  |
| 1992 | 31.7 (0.8) | 1462 | 4605645 |
| 1993 | 30.4 (0.8) | 1423 | 4684207 |
| 1994 | 31.9 (0.8) | 1514 | 4742583 |
| 1995 | 33.4 (0.8) | 1604 | 4809406 |
| 1996 | 33 (0.8) | 1607 | 4872302 |
| 1997 | 36.6 (0.9) | 1835 | 5007151 |
| 1998 | 36.5 (0.8) | 1869 | 5121456 |
| 1999 | 37.2 (0.8) | 1949 | 5244436 |
| 2000 | 33.1 (0.8) | 1777 | 5365165 |
| 2001 | 32.4 (0.8) | 1774 | 5483539 |
| 2002 | 31.6 (0.8) | 1769 | 5602644 |
| 2003 | 28.8 (0.7) | 1650 | 5730764 |
| 2004 | 27.8 (0.7) | 1631 | 5860116 |
| 2005 | 29 (0.7) | 1742 | 6001854 |
| 2006 | 28.7 (0.7) | 1766 | 6143196 |
| 2007 | 29.7 (0.7) | 1871 | 6298314 |
| 2008 | 31.2 (0.7) | 2016 | 6458452 |
| 2009 | 33.3 (0.7) | 2205 | 6626585 |
| 2010 | 31.9 (0.7) | 2168 | 6799933 |
| 2011 | 33 (0.7) | 2299 | 6962872 |
| 2012 | 35.2 (0.7) | 2507 | 7120060 |
| 50-59 y |  |  |  |
| 1992 | 20.1 (1.1) | 311 | 1550315 |
| 1993 | 18.8 (1.1) | 303 | 1609484 |
| 1994 | 18.3 (1.1) | 303 | 1658325 |
| 1995 | 21 (1.1) | 357 | 1702358 |
| 1996 | 21.2 (1.1) | 371 | 1747392 |
| 1997 | 21.3 (1.1) | 398 | 1867254 |
| 1998 | 25.1 (1.1) | 493 | 1962150 |
| 1999 | 23.4 (1.1) | 480 | 2054009 |
| 2000 | 21.7 (1) | 465 | 2146103 |
| 2001 | 20.4 (1) | 456 | 2236504 |
| 2002 | 21.9 (1) | 508 | 2318999 |
| 2003 | 18.9 (0.9) | 452 | 2387640 |
| 2004 | 19.2 (0.9) | 473 | 2467192 |
| 2005 | 19.2 (0.9) | 491 | 2553940 |
| 2006 | 18.9 (0.8) | 499 | 2641145 |
| 2007 | 19.4 (0.9) | 521 | 2682250 |
| 2008 | 19.5 (0.8) | 534 | 2737442 |
| 2009 | 20.6 (0.9) | 576 | 2791781 |
| 2010 | 20.5 (0.8) | 584 | 2845990 |
| 2011 | 20.3 (0.8) | 587 | 2888813 |
| 2012 | 24.3 (0.9) | 712 | 2926706 |
| 60-69 y |  |  |  |
| 1992 | 32.7 (1.5) | 452 | 1383959 |
| 1993 | 29.4 (1.5) | 402 | 1366505 |
| 1994 | 34.9 (1.6) | 470 | 1344859 |
| 1995 | 35.3 (1.6) | 471 | 1333223 |
| 1996 | 31.1 (1.5) | 411 | 1323631 |
| 1997 | 39.4 (1.7) | 518 | 1316029 |
| 1998 | 37.8 (1.7) | 497 | 1315830 |
| 1999 | 39.4 (1.7) | 521 | 1321886 |
| 2000 | 36.6 (1.7) | 486 | 1329284 |
| 2001 | 33.8 (1.6) | 455 | 1344762 |
| 2002 | 33.6 (1.6) | 462 | 1373648 |
| 2003 | 32.3 (1.5) | 460 | 1424945 |
| 2004 | 27.8 (1.4) | 409 | 1472476 |
| 2005 | 30.9 (1.4) | 468 | 1515469 |
| 2006 | 30.9 (1.4) | 482 | 1561719 |
| 2007 | 34.9 (1.4) | 580 | 1664157 |
| 2008 | 36.7 (1.4) | 643 | 1753471 |
| 2009 | 38.7 (1.4) | 715 | 1846077 |
| 2010 | 33.3 (1.3) | 646 | 1940255 |
| 2011 | 38.2 (1.4) | 774 | 2028374 |
| 2012 | 38.9 (1.4) | 820 | 2109458 |
| 70-79 y |  |  |  |
| 1992 | 42.2 (2) | 445 | 1054285 |
| 1993 | 41.7 (2) | 446 | 1070659 |
| 1994 | 45 (2) | 488 | 1084764 |
| 1995 | 46.1 (2) | 507 | 1100088 |
| 1996 | 47.5 (2.1) | 527 | 1110431 |
| 1997 | 52.5 (2.2) | 587 | 1118478 |
| 1998 | 49.3 (2.1) | 554 | 1124313 |
| 1999 | 54.1 (2.2) | 612 | 1131974 |
| 2000 | 46.2 (2) | 525 | 1136769 |
| 2001 | 45 (2) | 509 | 1130692 |
| 2002 | 43.6 (2) | 488 | 1120518 |
| 2003 | 40 (1.9) | 444 | 1111225 |
| 2004 | 37 (1.8) | 406 | 1097556 |
| 2005 | 39.4 (1.9) | 430 | 1090452 |
| 2006 | 40.2 (1.9) | 436 | 1083724 |
| 2007 | 39.5 (1.9) | 427 | 1081369 |
| 2008 | 43.4 (2) | 471 | 1084843 |
| 2009 | 45.4 (2) | 497 | 1094818 |
| 2010 | 47.6 (2.1) | 527 | 1106578 |
| 2011 | 46.4 (2) | 523 | 1126659 |
| 2012 | 50.4 (2.1) | 583 | 1156443 |
| 80+ y |  |  |  |
| 1992 | 40.8 (2.6) | 252 | 617086 |
| 1993 | 42.5 (2.6) | 271 | 637559 |
| 1994 | 38.6 (2.4) | 253 | 654635 |
| 1995 | 39.9 (2.4) | 269 | 673737 |
| 1996 | 43 (2.5) | 297 | 690848 |
| 1997 | 47.1 (2.6) | 332 | 705390 |
| 1998 | 45.2 (2.5) | 325 | 719163 |
| 1999 | 45.6 (2.5) | 336 | 736567 |
| 2000 | 40 (2.3) | 301 | 753009 |
| 2001 | 45.8 (2.4) | 353 | 771581 |
| 2002 | 39.4 (2.2) | 311 | 789479 |
| 2003 | 36.3 (2.1) | 293 | 806954 |
| 2004 | 41.7 (2.3) | 343 | 822892 |
| 2005 | 41.8 (2.2) | 352 | 841993 |
| 2006 | 40.6 (2.2) | 348 | 856608 |
| 2007 | 39.4 (2.1) | 343 | 870538 |
| 2008 | 41.7 (2.2) | 368 | 882696 |
| 2009 | 46.6 (2.3) | 417 | 893909 |
| 2010 | 45.3 (2.2) | 411 | 907110 |
| 2011 | 45.2 (2.2) | 415 | 919026 |
| 2012 | 42.3 (2.1) | 392 | 927453 |
| White women | |  |  |
| 1992 | 35.6 (1) | 1352 | 3797105 |
| 1993 | 33.7 (0.9) | 1295 | 3847020 |
| 1994 | 35.6 (1) | 1380 | 3876931 |
| 1995 | 37.1 (1) | 1451 | 3913085 |
| 1996 | 37.1 (1) | 1463 | 3945020 |
| 1997 | 40.7 (1) | 1642 | 4038125 |
| 1998 | 41.6 (1) | 1711 | 4109505 |
| 1999 | 42.4 (1) | 1775 | 4186570 |
| 2000 | 37.5 (0.9) | 1595 | 4258336 |
| 2001 | 36.8 (0.9) | 1593 | 4328972 |
| 2002 | 36 (0.9) | 1585 | 4400051 |
| 2003 | 32.7 (0.9) | 1466 | 4477858 |
| 2004 | 31.3 (0.8) | 1424 | 4556511 |
| 2005 | 33.3 (0.8) | 1548 | 4642997 |
| 2006 | 32.6 (0.8) | 1540 | 4728648 |
| 2007 | 33.8 (0.8) | 1633 | 4826625 |
| 2008 | 35.1 (0.8) | 1728 | 4929952 |
| 2009 | 37.7 (0.9) | 1896 | 5035115 |
| 2010 | 35.8 (0.8) | 1840 | 5145653 |
| 2011 | 37.1 (0.8) | 1950 | 5251056 |
| 2012 | 38.8 (0.9) | 2076 | 5349113 |
| Black women | |  |  |
| 1992 | 17.7 (2.1) | 70 | 395275 |
| 1993 | 19.6 (2.2) | 79 | 402427 |
| 1994 | 19.8 (2.2) | 81 | 409741 |
| 1995 | 22.7 (2.3) | 95 | 417971 |
| 1996 | 20.4 (2.2) | 87 | 425733 |
| 1997 | 27.3 (2.5) | 120 | 439289 |
| 1998 | 16.1 (1.9) | 73 | 453667 |
| 1999 | 19.8 (2.1) | 93 | 469920 |
| 2000 | 20.6 (2.1) | 100 | 485765 |
| 2001 | 18.1 (1.9) | 91 | 501720 |
| 2002 | 18.6 (1.9) | 96 | 517412 |
| 2003 | 20.1 (1.9) | 107 | 533564 |
| 2004 | 19.8 (1.9) | 109 | 549419 |
| 2005 | 17.6 (1.8) | 100 | 567539 |
| 2006 | 19.7 (1.8) | 116 | 587434 |
| 2007 | 21.2 (1.9) | 129 | 607234 |
| 2008 | 23.2 (1.9) | 145 | 625438 |
| 2009 | 26 (2) | 169 | 649775 |
| 2010 | 25.1 (1.9) | 169 | 672454 |
| 2011 | 24 (1.9) | 166 | 693048 |
| 2012 | 31.9 (2.1) | 228 | 714539 |
| Endometrioid Carcinoma of Uterus | | |  |
| 1992 | 8.9 (0.4) | 412 | 4605645 |
| 1993 | 11.1 (0.5) | 521 | 4684207 |
| 1994 | 16 (0.6) | 760 | 4742583 |
| 1995 | 20.6 (0.7) | 990 | 4809406 |
| 1996 | 24.4 (0.7) | 1189 | 4872302 |
| 1997 | 28.2 (0.8) | 1413 | 5007151 |
| 1998 | 32.3 (0.8) | 1655 | 5121456 |
| 1999 | 34 (0.8) | 1784 | 5244436 |
| 2000 | 34.8 (0.8) | 1867 | 5365165 |
| 2001 | 38 (0.8) | 2083 | 5483539 |
| 2002 | 39.8 (0.8) | 2230 | 5602644 |
| 2003 | 40.5 (0.8) | 2323 | 5730764 |
| 2004 | 41.1 (0.8) | 2408 | 5860116 |
| 2005 | 41.5 (0.8) | 2489 | 6001854 |
| 2006 | 43.6 (0.8) | 2678 | 6143196 |
| 2007 | 44.4 (0.8) | 2799 | 6298314 |
| 2008 | 46.5 (0.8) | 3006 | 6458452 |
| 2009 | 49.7 (0.9) | 3296 | 6626585 |
| 2010 | 51.4 (0.9) | 3494 | 6799933 |
| 2011 | 52.4 (0.9) | 3648 | 6962872 |
| 2012 | 52.2 (0.9) | 3715 | 7120060 |

| Table B. Calculated values. |  |  |  |  |
| --- | --- | --- | --- | --- |
|  |  |  |  |  |
| Tests of parallelism | Joinpoints | DF | Permutations | P |
| Breast Carcinoma, Ductal v Lobular | 2 | 5, 30 | 4500 | 0.002 |
| Ductal Carcinoma |  |  |  |  |
| All v 50-59 y | 2 | 5, 30 | 4500 | 0.003 |
| All v 60-69 y | 2 | 5, 30 | 4500 | < 0.001 |
| All v 70-79 y | 3 | 7, 26 | 4500 | 0.008 |
| All v 80+ y | 2 | 5, 30 | 4500 | 0.046 |
| 50-59 y v 60-69 y | 2 | 5, 30 | 4500 | < 0.001 |
| 50-59 y v 70-79 y | 3 | 7, 26 | 4500 | < 0.001 |
| 50-59 y v 80+ y | 2 | 5, 30 | 4500 | 0.06 |
| All v White women | 2 | 5, 30 | 4500 | < 0.001 |
| All v Black women | 2 | 5, 30 | 4500 | < 0.001 |
| White v Black women | 2 | 5, 30 | 4500 | < 0.001 |
| Lobular Carcinoma |  |  |  |  |
| All v 50-59 y | 2 | 5, 30 | 4500 | 0.16 |
| All v 60-69 y | 2 | 5, 30 | 4500 | 0.01 |
| All v 70-79 y | 2 | 5, 30 | 4500 | 0.29 |
| All v 80+ y | 2 | 5, 30 | 4500 | 0.06 |
| 50-59 y v 60-69 y | 2 | 5, 30 | 4500 | 0.44 |
| 50-59 y v 70-79 y | 2 | 5, 30 | 4500 | 0.28 |
| 50-59 y v 80+ y | 2 | 5, 30 | 4500 | 0.52 |
| All v White women | 2 | 5, 30 | 4500 | 0.009 |
| All v Black women | 2 | 5, 30 | 4500 | 0.0007 |
| White v Black women | 2 | 5, 30 | 4500 | 0.002 |
|  |  |  |  |  |
| Total Hip Replacement, Men v Women | 3 | 7, 14 | 4500 | 0.11 |
| Femoral Neck Fracture, Men v Women | 0 | 1, 26 | 4500 | 0.24 |
| Myocardial Infarction, Men v Women | 2 | 5, 18 | 4500 | 0.1 |
| Cerebrovascular Accident, Men v Women | 1 | 3, 22 | 4500 | 0.19 |
|  |  |  |  |  |
| Annual Percent Changes | Period | APC | 95% CI | P |
| HRT Prescriptions | 1996-2001 | 7.9 | 4.5, 11.5 | < 0.001 |
|  | 2001-2004 | -26.2 | -34, -17.5 | < 0.001 |
|  | 2004-2012 | -10.3 | -12.2, -8.4 | < 0.001 |
| Ductal Breast Carcinoma | 1992-1999 | 1.2 | 0.5, 1.9 | < 0.001 |
|  | 1999-2003 | -4.5 | -6.8, -2.2 | < 0.001 |
|  | 2003-2012 | 1.1 | 0.7, 1.5 | < 0.001 |
| Lobular Breast Carcinoma | 1992-1999 | 2.6 | 1.3, 3.9 | < 0.001 |
|  | 1999-2004 | -5.6 | -8.3, -2.7 | < 0.001 |
|  | 2004-2012 | 2.9 | 2, 3.9 | < 0.001 |
| Endometrioid Carcinoma | 1992-1995 | 33.3 | 23, 44.4 | < 0.001 |
|  | 1995-1998 | 17.2 | 5.5, 30.1 | 0.001 |
|  | 1998-2012 | 3.5 | 3.1, 3.8 | < 0.001 |
|  |  |  |  |  |
| Ductal Carcinoma Subgroups |  |  |  |  |
| 50-59 y | 1992-1999 | 2.5 | 1.6, 3.4 | < 0.001 |
|  | 1999-2003 | -4.4 | -7.1, -1.5 | 0.001 |
|  | 2003-2012 | 0.4 | -0.2, 0.9 | 0.13 |
| 60-69 y | 1992-2000 | 1.4 | 0.5, 2.3 | < 0.001 |
|  | 2000-2003 | -4.6 | -11.8, 3.1 | 0.19 |
|  | 2003-2012 | 1.5 | 0.9, 2.2 | < 0.001 |
| 70-79 y | 1992-1994 | -2.8 | -9.7, 4.7 | 0.4 |
|  | 1994-1998 | 2.9 | -0.7, 6.7 | 0.07 |
|  | 1998-2003 | -4.6 | -6.8, -2.4 | < 0.001 |
|  | 2003-2012 | 1.9 | 1.2, 2.6 | < 0.001 |
| 80+ y | 1992-1998 | 0.6 | -0.5, 1.7 | 0.26 |
|  | 1998-2003 | -3.2 | -5.1, -1.3 | < 0.001 |
|  | 2003-2012 | 0.7 | 0.2, 1.2 | 0.006 |
| White Race | 1992-1999 | 1.2 | 0.5, 1.9 | < 0.001 |
|  | 1999-2003 | -4.7 | -7.2, -2.2 | < 0.001 |
|  | 2003-2012 | 0.9 | 0.4, 1.4 | < 0.001 |
| Black Race | 1992-1998 | 2.3 | 0.5, 4.2 | 0.006 |
|  | 1998-2001 | -3.3 | -12.2, 6.5 | 0.45 |
|  | 2001-2012 | 1.6 | 1, 2.2 | < 0.001 |
|  |  |  |  |  |
| 50-59 y | 1992-1998 | 4.1 | 0.9, 7.4 | 0.005 |
|  | 1998-2006 | -3 | -5, -1 | 0.002 |
|  | 2006-2012 | 3.7 | 1.2, 6.2 | 0.001 |
| 60-69 y | 1992-1999 | 3.2 | 0.2, 6.3 | 0.02 |
|  | 1999-2004 | -5.1 | -11.5, 1.7 | 0.1 |
|  | 2004-2012 | 3.5 | 1.4, 5.7 | < 0.001 |
| 70-79 y | 1992-1999 | 3.3 | 2, 4.7 | < 0.001 |
|  | 1999-2004 | -6.7 | -9.6, -3.8 | < 0.001 |
|  | 2004-2012 | 3.8 | 2.7, 4.9 | < 0.001 |
| 80+ y | 1992-2012 | 0.2 | -0.4, 0.7 | 0.48 |
| White Race | 1992-1999 | 2.9 | 1.6, 4.3 | < 0.001 |
|  | 1999-2004 | -5.4 | -8.3, -2.5 | < 0.001 |
|  | 2004-2012 | 2.6 | 1.6, 3.6 | < 0.001 |
| Black Race | 1992-2005 | -0.8 | -2.8, 1.2 | 0.39 |
|  | 2005-2012 | 6.5 | 2.4, 10.8 | 0.001 |
|  |  |  |  |  |
| Total Hip Replacement | 1996-2000 | 0.5 | -2.9, 4 | 0.77 |
|  | 2000-2004 | 6.8 | 1.4, 12.4 | 0.006 |
|  | 2004-2007 | -3.4 | -12.4, 6.6 | 0.46 |
|  | 2007-2010 | 13.3 | 3.9, 23.6 | 0.002 |
| Femoral Neck Fracture | 1996-2010 | -3.6 | -4.7, -2.5 | < 0.001 |
| Myocardial Infarction | 1996-2002 | 0 | -1.8, 1.8 | 0.99 |
|  | 2002-2007 | -7.7 | -10.5, -4.8 | < 0.001 |
|  | 2007-2010 | 1.8 | -8.2, 12.9 | 0.72 |
| Cerebral Infarction | 1996-2001 | -4.6 | -7.8, -1.4 | 0.003 |
|  | 2001-2010 | 2.5 | 1, 4 | < 0.001 |
|  |  |  |  |  |
| Annual Percent Changes | Period | AAPC | 95% CI | P |
| HRT Prescription | 2001-2012 | -14.9 | -17.4, -12.4 | < 0.001 |
| Ductal Breast Carcinoma | 2001-2012 | 0.1 | -0.4, 0.6 | < 0.001 |
| Lobular Breast Carcinoma | 2001-2012 | 0.5 | -0.4, 1.5 | < 0.001 |
| Endometrioid Carcinoma | 2001-2012 | 3.5 | 3.1, 3.8 | < 0.001 |
| Total Hip Replacement | 2001-2010 | 5.3 | 0.9, 10 | < 0.001 |
| Femoral Neck Fracture | 2001-2010 | -3.6 | -4.7, -2.5 | < 0.001 |
| Myocardial Infarction | 2001-2010 | -3.8 | -7.2, -0.2 | < 0.001 |
